# An estimation of the endoscopist's musculoskeletal injury risk for right and left lateral decubitus positions during colonoscopy: a field-based ergonomic study

**DOI:** 10.1186/s12891-023-06606-4

**Published:** 2023-06-10

**Authors:** Maxim Landry, Sarah Mackey, Intekhab Hossain, Nicholas Fairbridge, Alison Greene, Mark Borgaonkar, Kimberley Cullen, David Pace, Diana De Carvalho

**Affiliations:** 1 Faculty of Medicine, Memorial University, St. John’s, NL Canada; 2 Faculty of Medicine, Dalhousie University, Halifax, NS Canada; 3 School of Human Kinetics and Recreation, Memorial University, St. John’s, NL Canada

**Keywords:** Ergonomics, Colonoscopy, Left Lateral decubitus, Right lateral decubitus, Musculoskeletal injury

## Abstract

**Background:**

Colonoscopy exposes endoscopists to awkward postures and prolonged forces, which increases their risk of musculoskeletal injury. Patient positioning has a significant impact on the ergonomics of colonoscopy. Recent trials have found the right lateral decubitus position is associated with quicker insertion, higher adenoma detection rates, and greater patient comfort compared to the left lateral decubitus position. However, this patient position is perceived as more strenuous by endoscopists.

**Methods:**

Nineteen endoscopists were observed performing colonoscopies during a series of four-hour endoscopy clinics. Durations of each patient position (right lateral decubitus, left lateral decubitus, prone, and supine) were recorded for all observed procedures (*n* = 64). Endoscopist injury risk was estimated by a trained researcher for the first and last colonoscopies of the shifts (*n* = 34) using Rapid Upper Limb Assessment (RULA), an observational ergonomic tool that estimates risk of musculoskeletal injury by scoring postures of the upper body and factors such as muscle use, force, and load. The total RULA scores were compared with a Wilcoxon Signed-Rank test for patient position (right and left lateral decubitus) and time (first and last procedures) with significance taken at *p* < 0.05. Endoscopist preferences were also surveyed.

**Results:**

The right lateral decubitus position was associated with significantly higher RULA scores than the left lateral decubitus position (median 5 vs. 3, *p* < 0.001). RULA scores were not significantly different between the first and last procedures of the shifts (median 5 vs. 5, *p* = 0.816). 89% of endoscopists preferred the left lateral decubitus position, primarily due to superior ergonomics and comfort.

**Conclusion:**

RULA scores indicate an increased risk of musculoskeletal injury in both patient positions, with greater risk in the right lateral decubitus position.

## Introduction

Gastrointestinal (GI) endoscopy is an essential tool in the screening, diagnosis, and treatment of numerous GI conditions [1–4]. For the physician, it involves repeated pinching and gripping movements, as well as unnatural hand, wrist, elbow, shoulder, and neck positions, increasing musculoskeletal injury (MSI) risk with cumulative exposures [5–7]. According to various reports, 39—89% of endoscopists develop occupational musculoskeletal pain or injuries during their careers [1]. One particular survey of 684 endoscopists revealed that 53% had experienced a MSI perceived to be related to endoscopy [7]. Some of the most commonly reported repetitive-use injuries amongst endoscopists include: chronic neck strain, carpal tunnel syndrome, De Quervain’s Tenosynovitis (also known as "Colonoscopist’s Thumb"), and low back pain [8, 9]. Right upper extremity MSIs have been reported to result from torquing the colonoscope, while left upper extremity MSIs have been reported to result from turning control dials, particularly in the case of stiff dials and lengthy procedures [8, 9]. Neck and back pain are primarily the results of fixed, awkward monitor placement, and knee pain has been reported from pivoting to see the monitor [10]. Elevated risk of MSI has been demonstrated in some cohorts of endoscopists, including females, suggested to be due to decreased ability to generate force, and novices, potentially because of their increased procedure times resulting in increased cumulative exposure [11, 12]. In addition, higher procedural volume (> 20 patients/week and > 16 h/week), is associated with greater MSI risk [13]. MSIs can adversely affect physicians’ abilities to perform these procedures and result in lost-work-time and diminished quality of life [11].

Despite the ergonomic challenges of endoscopy and the implications for MSIs, a literature review of occupational hazards for endoscopists revealed that ergonomics remains a neglected aspect of endoscopy training and practice [14, 15]. In a survey of gastroenterology fellows, 72% responded that they did not feel they had received any formal training on preventing overuse injuries. In that same study, 27% responded “yes” or “maybe” to sustaining MSIs related to endoscopy, while 67% felt it was likely that they would sustain endoscopy-related MSIs over the course of their careers [16]. A recent study showed that a simulation-based ergonomics training curriculum is associated with reduced risk of MSI during colonoscopy [12]. Subsequently, the American Society for Gastrointestinal Endoscopy (ASGE) published a core curriculum for ergonomics in endoscopy which discusses technical factors including appropriate body position and room setup during endoscopy, proper technique for holding and handling the endoscope, and various strategies (e.g., turning patients, torque steering) to minimize musculoskeletal load [17].

Patient position during colonoscopy may also impact MSI risk. Though data is conflicting, the right lateral decubitus (RLD) position has been found to decrease time to reach the cecum by 30% and increase patient comfort compared to the left lateral decubitus (LLD) position [18]. However, this patient position requires more extreme postures on the part of the endoscopist to reach over the patient, potentially increasing MSI risk. Endoscopists have reported anecdotally that the RLD position is perceived to be more strenuous than the LLD position; however, this has yet to be studied formally. Therefore, the purpose of this investigation is to determine whether there is a difference in MSI risk for the endoscopist when performing colonoscopies with patients in the RLD and LLD positions.

## Materials and methods

### Participants

A convenience sample of nineteen endoscopists were recruited from the Health Sciences Centre and St. Clare’s Mercy Hospital (St. John’s NL). All endoscopists had previously completed a colonoscopy skills improvement course where optimal bed, stack, patient, and monitor position were discussed. Appropriate posture, hand position, and scope handling were also discussed, and feedback was given during and after the performance of colonoscopies. Informed consent was obtained from each clinician and their patients. This study was approved by the local Health Ethics Research Board (HREA # 2022.059).

### Rapid Upper Limb Assessment (RULA)

The Rapid Upper Limb Assessment (RULA) tool was utilized to assess the practitioners’ MSI risk with patients in the RLD and LLD positions [19]. This observational tool is a snapshot in time and involves the rater identifying and scoring the "worst" upper body posture adopted by a worker while considering factors such as muscle use, force, and load. The minimum RULA score is 1 and the maximum RULA score is 7. As the score increases, the risk of MSI increases. The score provides a prompt that indicates whether the posture requires no action (1–2), potential action (3–4), further investigation and change in the near future (5–6) and immediate change needed (7 +).

For this study, a trained and experienced rater performed all RULA assessments on the endoscopists’ right upper extremities by standing perpendicular to them with an unobstructed view to minimize errors in estimating body position. The tool took approximately three minutes to complete per posture and did not interfere with the colonoscopy procedure. RULA assessments were performed on the first and last colonoscopy of the shift only.

### Patient position exposure

To characterize the patient postures that the endoscopists were exposed to throughout the collection period, the duration of each patient position for all colonoscopies during the shift were timed with a stopwatch and recorded by a researcher in the room. The stopwatch was started when the procedure had begun, or the patient had settled in a new position and the examination restarted, and stopped when the patient was repositioned, or the procedure was completed.

### Endoscopist questionnaire

At the end of each clinic, clinicians were given a questionnaire assessing their preferred patient position and their perception of the musculoskeletal demands in both positions (RLD and LLD). The first question included a 5-point Likert scale ranging from “strongly disagree” to “strongly agree” with the statement “The patient position at the start of the procedure today is my preferred position for this procedure.” Next, the following questions were asked: (1) Which patient position do you prefer? (Circle one: RLD/LLD), (2) Considering your most preferred patient position, what do you like about performing this procedure with the patient in this position? (Free comment text, no limit) and (3) Considering your least preferred patient position, what do you dislike about performing this procedure with the patient in this position? (Free comment text, no limit).

### Data collection

**Fig. 1:**
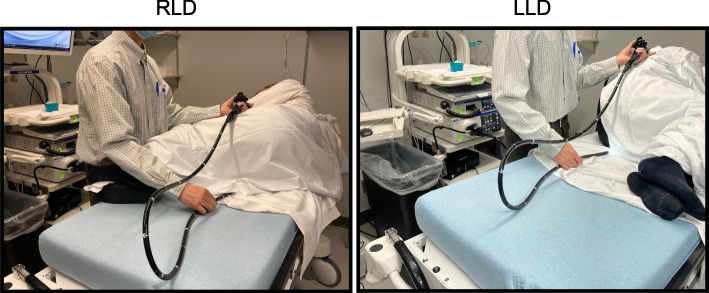
Representative photos of the right lateral decubitus (RLD), and left lateral decubitus (LRLD) postures examined in this study

The clinicians were observed performing elective colonoscopies throughout the duration of a series of four-hour shifts. Demographic information for each clinician (height, weight, glove size, years of experience) were collected. Each practitioner was instructed to begin the first (*n* = 30) and last (*n* = 25) colonoscopy of the shift with the patient in the RLD position, after which they were permitted to position the patient as preferred. RULA scores of the first and last colonoscopy were taken. Specifically, a trained observer used the RULA tool to assess the endoscopist’s right upper extremity while the patient was in either the LLD (*n* = 22) or RLD (*n* = 33) position. Additionally, the time each patient spent in each position (LLD, RLD, supine and prone) was recorded for all procedure during the shift. At the end of the shift, the endoscopists were given the questionnaire to complete. Representative photos of each patient position are included in Fig. 1. Retrospectively, patient demographics (age, sex, height, weight) for all colonoscopies conducted during the study shifts were extracted to characterize the patient population.

### Statistics

The dependent measures for this investigation were the RULA scores for each patient position (RLD/LLD) during the first and last procedures of the endoscopists’ shifts. SPSS statistical package version 27 (IBM Corp. Released 2020. IBM SPSS Statistics for Windows, Version 27.0. Armonk, NY: IBM Corp) was used for all statistical analyses. The total RULA scores were compared with a Wilcoxon Signed-Rank test for patient position (RLD and LLD) and time (first and last procedures) with significance taken at *p* < 0.05. Ordinal regression was performed to assess the influence of patient and practitioner characteristics on RULA scores. Non-parametric tests were selected based on the ordinal nature of RULA scores. All data was also scanned for ceiling/floor limitations and RULA scores were excluded from statistical analysis when there was no corresponding score during the same procedure in the opposite patient position (RLD vs LLD) and no corresponding score for the same patient position during the other RULA-scored procedure of the shift (first vs last).

## Results

### Participant characteristics

Nineteen endoscopists completed this study. This cohort represents 76% of the eligible participants in our facilities and included 12 males, 7 females, 95% right hand dominant, average experience 12 (sd 11) years, average height 1.73 (sd 0.10) m, average weight 79.3 (sd 10.6) kg, average BMI 26.3 (sd 2.8) kg/m^2^, median glove size 7.5 (MAD 0.5). Participating clinicians declared no current or recent musculoskeletal pain or injuries on the day of data collection.

### Characterizing the observed procedures

The colonoscopies of 64 different patients were observed for this study. Thirty-seven patients were included in either the first or last colonoscopy of the shift (with RULA scores and posture timing measured). There were no statistical differences for age/height/weight/BMI of the patients in the first and last exam so average characteristics are presented: 19 male, 18 female, average age 61 (sd 11.9) years, average height 1.68 (sd 0.09) m, average weight 81.9 (sd 22.9) kg, average BMI 28.9 (sd 6.65) kg/m^2^. Twenty-seven patients were included in the intervening colonoscopy exams (posture timing measured): 9 male, 18 female, average age 65 (sd 13) years, average height 1.65 (sd 0.09) m, average weight 77.3 (sd 14.5) kg, average BMI 28.3 (sd 4.6) kg/m^2^. The average time patients spent in the RLD position was 7.30 (sd 4.53) minutes per procedure and in the LLD position was 9.18 (sd 7.40) minutes.

### RULA scores

**Fig. 2:**
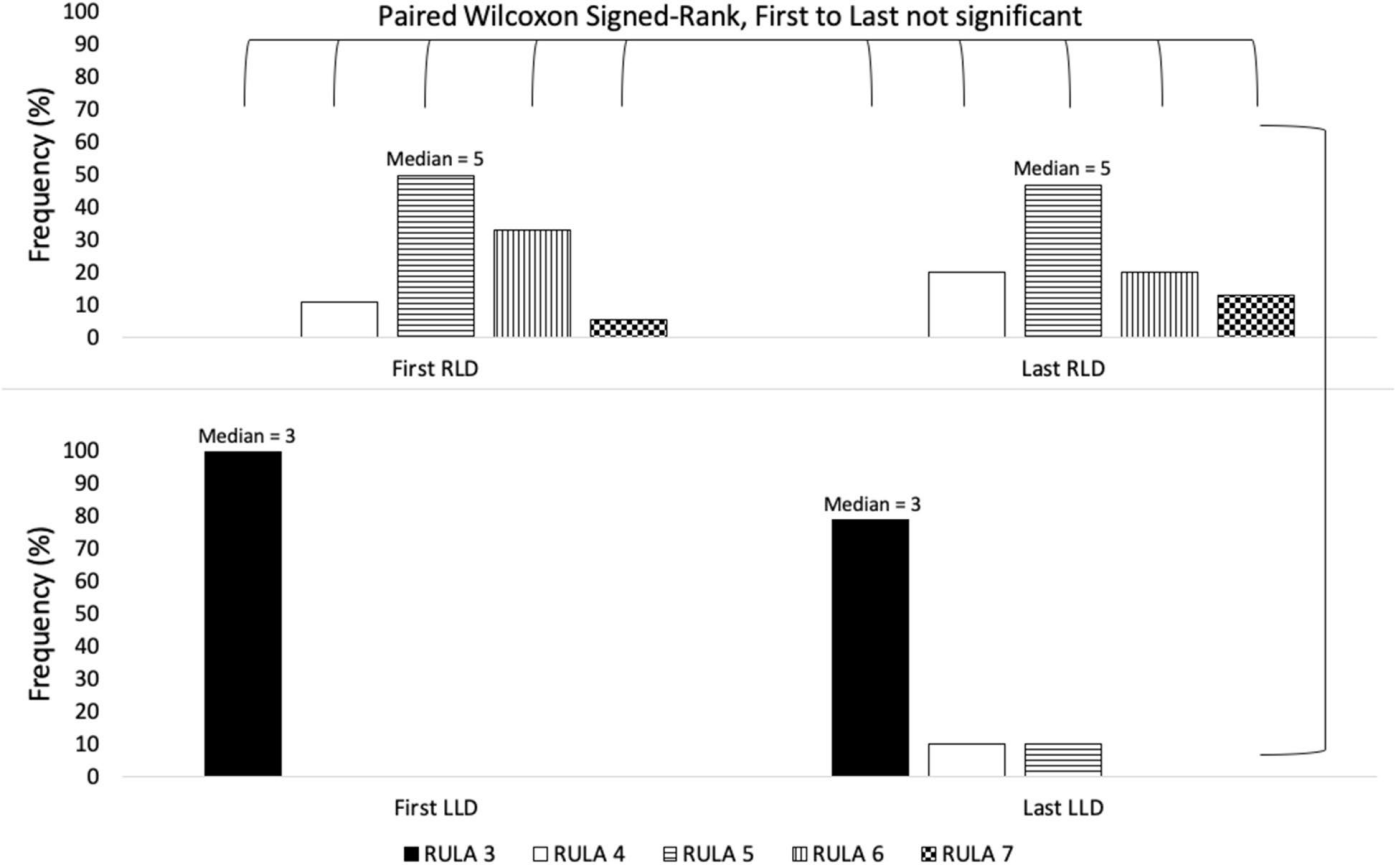
Frequency distribution of RULA scores. Patients in the right and left lateral decubitus positions (RLD/LLD) during the first and last procedures of endoscopy shifts (First/Last). Wilcoxon Signed-Rank tests were conducted to compare ranked scores between RLD/LLD and between first/last procedures. The LLD patient position demonstrated a protective effect to physician ergonomics as suggested by RULA scores (median 5 vs. 3, p < 0.001, z = -4.029)

The RLD patient position was associated with significantly higher RULA scores than the LLD position (median 5 vs. 3, *p* < 0.001, z = -4.029). See Fig. 2. RULA scores were not significantly different between the first and last procedures of each shift (median 5 vs. 5, *p* = 0.816, z = -0.233). The frequency distribution of RULA scores with patients in the RLD and LLD positions during the first and last procedures of the shifts is shown in Fig. 2. The strong protective effect of the LLD position masked any variation that may have been associated with patient or endoscopist characteristics. RULA scores did however show more variation with patients in the RLD position. Thus, only RLD RULA scores were used in an ordinal regression to examine patient and practitioner characteristics. RLD RULA scores showed no relationship to clinician glove size, sex, age, or experience. However, an increase in clinician weight was associated with increased RULA scores, while an increase in clinician height was associated with decreased RULA scores with the patient in the RLD position (Table 1). Patient characteristics (height, weight, BMI, age, sex) did not have any influence on RULA score.

**Table 1:** Ordinal regression examining the influence of practitioner characteristics on RULA scores

	Estimate	Std. Error	Wald	df	Sig	Relative Odds	Lower Bound	Upper Bound
Height (m)	-136	56.9	5.68	1	0.017	1.23e^−59^	4.38e^−108^	3.46e^−11^
Weight (kg)	1.25	0.573	4.77	1	0.029	3.49	1.14	10.7
BMI (kg/m^2^)	-3.93	1.71	5.31	1	0.021	0.020	6.96e^−4^	0.556
Glove size	NS							
Experience	NS							
Sex	NS							
Handedness	NS							
Arm with most discomfort	NS							

*NS* Not Significant, *Std. Error* Standard error, *df* degrees of freedom, *sig.* significance

### Endoscopist preferences

A summary of endoscopist discomfort and preferred patient position is included in Table 2. 47% (*n* = 9) of endoscopists noted their left arm causing them the most discomfort while performing the procedure, 32% (*n* = 6) felt it was their right arm, and 21% (*n* = 4) did not favor one side over the other. 89% (*n* = 17) chose LLD as their preferred position of the two, while 5% (*n* = 1) chose RLD. 5% (*n* = 1) were undecided. All clinicians who preferred the LLD position (*n* = 17) cited physical challenges associated with leaning over the patient in the RLD position. Back pain (*n* = 3), upper body pain (*n* = 2), calf pain (*n* = 1), increased difficulty torquing the scope (*n* = 2), and an inability to maintain a neutral wrist posture (*n* = 1) were also cited as factors contributing to their non-preference of the RLD position. Comments favoring the RLD position mentioned quicker time to reach the splenic flexure (*n *= 1) and ease of navigating difficult sigmoid colons (*n* = 1).

**Table 2:** Endoscopist preference of patient position and arm with most discomfort

**Variable**	**Categories**	**Number of Participants**
Arm with most discomfort	Left	*n* = 9 (49%)
	Right	*n* = 6 (32%)
	Neither	*n* = 4 (21%)
Preferred patient position	LLD	*n* = 17 (89%)
	RLD	*n* = 1 (5%)
	Undecided	*n* = 1 (5%)

## Discussion

Analysis of RULA scores suggests that the performance of colonoscopy with patients in both the RLD and LLD positions is associated with an increased MSI risk, though the RLD position was found to pose a greater risk (median 5 vs. 3, *p* < 0.001, z = -4.029). Furthermore, the LLD position was protective against variation in RULA scores associated with endoscopist characteristics, which was seen with RLD patient positioning. Given that colonoscopy is usually performed with a LLD patient position, which favors endoscopist comfort, many endoscopists will likely be reluctant to move patients into the RLD position. This is despite the potential benefits of RLD positioning, including improved mucosal visualization in the cecum [20] and left colon [21], higher adenoma detection rates in the left colon [22], and possibly quicker insertion times [18].

The performance of colonoscopy with the patient in the RLD position makes the initial rectal exam and colonoscope insertion more awkward for the endoscopist. Visualization is impaired and the endoscopist must reach over the patient’s hips to perform these maneuvers. In addition, the RLD position forces the endoscopist to reach forward given that the anus is further away from the side of endoscopy table. This results in flexion of the endoscopist’s torso, extension of the arms, and extension of the neck to maintain a good view of the monitor, potentially leading to discomfort and injury. Heavier endoscopists are even more hindered by these maneuvers, while taller endoscopists are less so, as shown in Table 1. These observations are consistent with questionnaire responses, where most physicians (89%) reported a preference for the LLD position over the RLD position. This preference was also reflected in the duration that endoscopists had patients in each position, which was longer for the LLD position (mean 9.18 min) than the RLD position (mean 7.30 min). We note that the preference for the LLD position also aligns with lower RULA scores; however, given the sample size we were not able to explore this correlation statistically.

The RULA scores in this study, with median scores ranging from of 3 to 5, were much lower than those noted in the simulation-based study by Khan et al. [12]. This can be explained by the fact that the endoscopists in our study were more experienced and had all received ergonomics training during a hands-on colonoscopy skills improvement course. The creators of RULA recommend further investigation into the activity with possible implementation of changes when a mean RULA score of 3–4 is obtained, which is where the endoscopists scored with the patient in the LLD position. The recommendation associated with a RULA score of 5–6, where the endoscopists scored with the patient in the RLD position, is for further investigation and timely changes [19]. There is no established minimally important difference for RULA scores; however, practically we would interpret our result to indicate that the RLD position scores are of greater concern and injury risk than those found in the LLD position.

Limitations to this study include its small sample size, non-blinding of the assessor and a lack of randomization of patient positioning. Although our sample size was relatively small for this investigation, we were able to recruit 19 out of 25 available clinicians from facilities which represents the majority of our local clinician population (76%). The sample also appears to have achieved acceptable power given that statistically significant difference in RULA scores were observed between the LLD and RLD patient positions. While the results represent our provincial population well, they will have limited generalizability overall. We hope this study will be replicated in different regions and with larger samples in the future. We were not able to randomize patient posture in this study because when piloting the data collection, we noted that physicians were often avoiding the RLD position altogether. Therefore, positions were fixed for the first and last procedures to ensure both postures were utilized with minimum disruption to the patient encounter. Additionally, the number of procedures per shift and intervening procedures, such as gastroscopies and sigmoidoscopies, between colonoscopies could not be standardized due to logistical challenges. This negated the possibility of accurately assessing the accumulation of fatigue during a shift. An in-depth biomechanical analysis of practitioner posture and muscular demand in a laboratory-controlled study of the simulated patient positions would further our understanding of this issue and is underway.

This is the first study to evaluate the ergonomics of RLD patient positioning during colonoscopy. The results suggest that the colonoscopy procedure involves MSI risk, and that patient positioning is an important factor influencing the ergonomics of colonoscopy. From an ergonomic standpoint for endoscopists, performing colonoscopies with the patient in the RLD position appears to be much less comfortable than the LLD position and poses a greater MSI risk. The RULA scores for the RLD in this study indicate that this patient position should only be used at specific points in the procedure to optimize visualization or to aid in colonoscope insertion. While a larger cohort of patients with control of potential confounding variables would reaffirm these findings, further efforts need to be made to improve the ergonomics of the procedure. Even though the endoscopists in this study knew about the importance of ergonomics and had received instruction to improve their technique, their RULA scores were still higher than desirable. Potential solutions include a more ergonomic colonoscope design and/or a fully automated colonoscopy insertion device. Also, the avoidance of deep sedation, which is commonly used in many centers, facilitates position change, which may limit injury to those responsible for moving patients during the procedure [23]. Future work should expand upon the existing practical knowledge for the improvement of the ergonomics of colonoscopy.

## Data Availability

The full dataset will be made available upon request.

## Abbreviations

RULA: Rapid Upper Limb Assessment
GI: Gastrointestinal
MSI: Musculoskeletal Injury
ASGE: American Society for Gastrointestinal Endoscopy
RLD: Right Lateral Decubitus
LLD: Left Lateral Decubitus
NL: Newfoundland and Labrador
sd: Standard Deviation
BMI: Body Mass Index
m: Metres
kg: Kilograms
MAD: Mean Absolute Deviation
NS: Not Significant
Std. Error: Standard Error
df: Degrees of Freedom
sig.: Significance
min: Minutes
